# Protein post-translational modifications: *In silico* prediction tools and molecular modeling

**DOI:** 10.1016/j.csbj.2017.03.004

**Published:** 2017-03-31

**Authors:** Martina Audagnotto, Matteo Dal Peraro

**Affiliations:** Institute of Bioengineering, School of Life Sciences, École Polytechnique Fédérale de Lausanne (EPFL), Lausanne, Switzerland; Swiss Institute of Bioinformatics (SIB), Lausanne, Switzerland

## Abstract

Post-translational modifications (PTMs) occur in almost all proteins and play an important role in numerous biological processes by significantly affecting proteins' structure and dynamics. Several computational approaches have been developed to study PTMs (*e.g.*, phosphorylation, sumoylation or palmitoylation) showing the importance of these techniques in predicting modified sites that can be further investigated with experimental approaches. In this review, we summarize some of the available online platforms and their contribution in the study of PTMs. Moreover, we discuss the emerging capabilities of molecular modeling and simulation that are able to complement these bioinformatics methods, providing deeper molecular insights into the biological function of post-translational modified proteins.

## 1 Introduction

Post-translational modifications (PTMs) occur on a large number of proteins de facto increasing the actual complexity of the proteome. PTMs consist in a covalent modification of amino acids of the primary protein sequence [1] and have the effect to create a much larger array of possible protein species. In response to specific physiological requirements, PTMs play a crucial role in regulating many biological functions [2], such as protein localization in the cell [3], [4], protein stability [5], and regulation of enzymatic activity [6]. To date more than 90,000 individual PTMs were detected using biochemical and biophysical analyses [7]. In particular, it was observed that almost 5% of the human genome encodes enzymes in charge of catalyzing reactions leading to PTMs [8], highlighting once more the importance of these chemical modifications of the proteome.

Enzymes often are responsible for regulating these chemical modifications in proteins, as in the case of phosphorylation, acetylation, methylation, carboxylation or hydroxylation [9]. For instance, protein kinases can phosphorylate a given protein target to induce a signaling cascade, while this PTM can be further removed by specific protein phosphatases. These enzymes are found indeed in important signaling pathways, like G-protein [9], [10] and Wnt signaling [11], [12]. On the other side, PTMs not induced by specific enzymes (*e.g.*, carbonylation or oxidations) were observed to be responsible of non-specific protein damage involved in neurodegenerative diseases, cancer and diabetes [13], [14], [15].

During the past 30 years, experimental techniques used for mapping and quantifying PTMs have seen an impressive progress. In particular, liquid chromatography (LC) with mass-spectrometry (MS) protein-based analysis allowed the detection of thousands of PTMs across entire proteomes [16]. The study of PTMs in their biological context was achieved thanks to advancements in fluorophore chemistry, fluorescence spectrometry, and peptide and antibody synthesis [17]. However, the identification and characterization of PTMs are still limited by the poor knowledge of the underlying enzymatic reactions and their final effects on protein stability and dynamics. In this context, *in silico* methods, often based on the current knowledge of PTMs, are a promising strategy to perform preliminary analysis and prediction that can guide further *in vivo* and *in vitro* experiments, leading to expand our understanding of the role of PTMs in cellular processes.

In this review, we provide an overview of some of the existing computational approaches used to study the most common PTMs, which we classified based on the covalent attachment of (i) small chemical groups, (ii) lipids or (iii) small proteins to the main peptide chain. Most of these tools are presented as online webservers, providing a user-friendly interface for PTM site identification. Although in this review we mainly focus our attention on this kind of resources, standalone software, like for instance PEAKS PTM [18], GlycoMaster [19] or MODa [20], are also available but won't be covered here.

## 2 Covalent attachment of small chemical groups

### 2.1 Phosphorylation

**Fig. 1:**
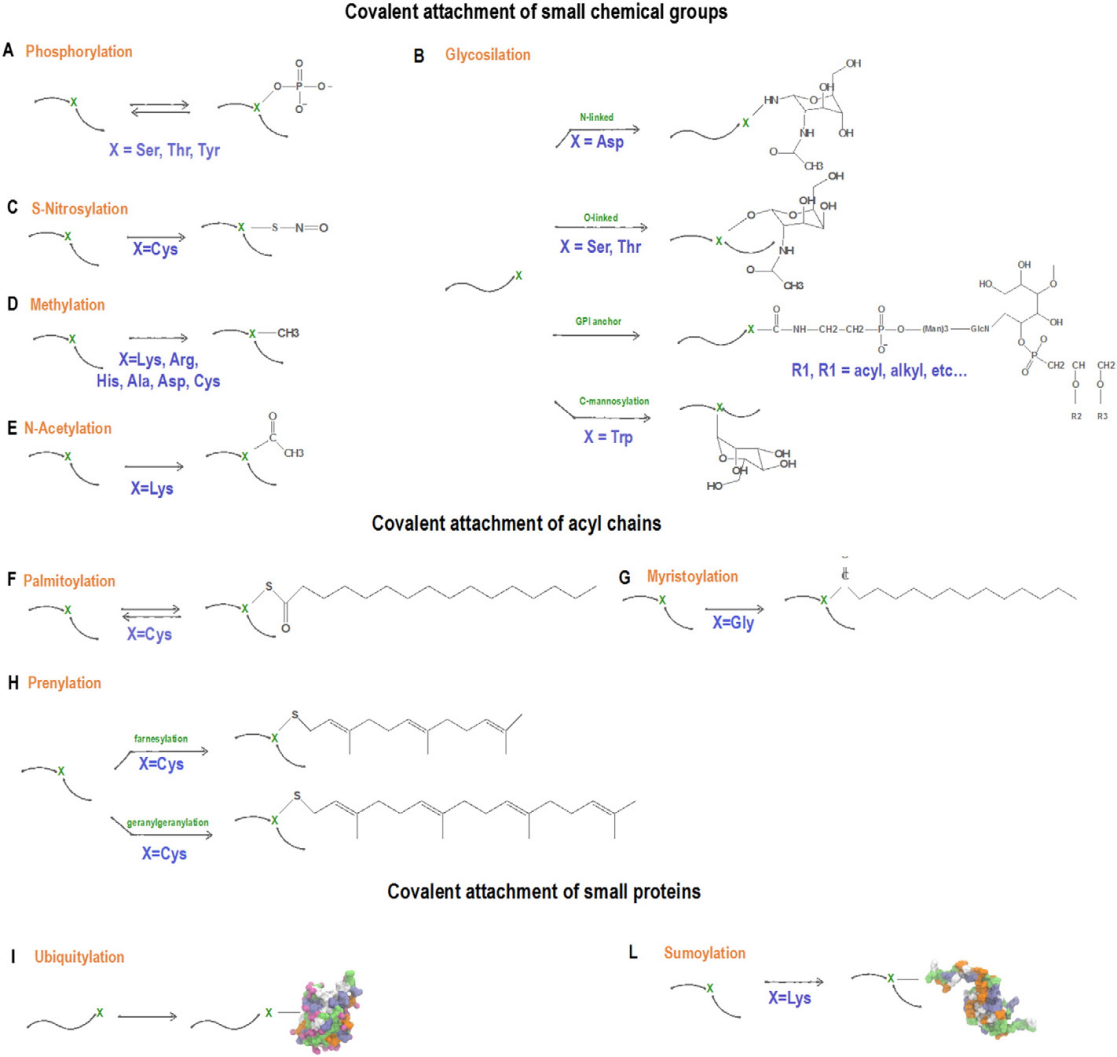
Schematic representation of PTMs discussed in this review.

Phosphorylation is the most studied PTM that involves the covalent addition of a small chemical group [21]. It is a reversible enzymatic reaction, which consists in the attachment of a phosphate group to the side chain of an arginine, lysine, histidine, tyrosine, serine or threonine residue [22] (Fig. 1A). It plays a key role in almost every cellular process, including metabolism, division, organelle trafficking, membrane transport, immunity, learning and memory [23], [24], and function of target proteins [25]. It can activate [26], [27] and inhibit [28], [29] enzyme activity through allosteric conformational changes, facilitate the recognition of other proteins [30], [31], [32], [33], promote protein-protein association [34], [35], [36] or dissociation [37] and also induce order-to-disorder transition [38], [39] (Table 2).

It was estimated that 30% of the total proteome is phosphorylated at least at one residue [40], [41]. However, this simple switch mechanism is in reality more complex since multiple enzymes can act on multiple sites of the same protein creating a highly connected network of interactions and modifications. For example, it was shown by high-resolution mass spectrometry that 37,248 phosphorylation sites are present on 5705 proteins in adipocyte cells [42]. Other phospho-proteomic analyses demonstrated that proteins, on average, could be phosphorylated on at least five different sites, although these results could suffer from biases coming from high stoichiometry of the complexes [43], [44], [45].

Advances in mass spectrometry, both in terms of speed and sensitivity, allowed identifying and quantifying thousands of phosphorylation sites in different species [42], [45]. The conservation of the functional phosphorylation sites in species like mice, rats and flies is a feature used by biologists for selecting specific sites of interest for functional characterization. Therefore, mapping the phosphorylation sites on proteins is an important step in order to understand the catalytic process and the effects of signal transduction events. However, it is still in general difficult to identify specific phosphorylation sites. *In silico* predictions play an important role in this field. Several methods (Table 1) were implemented in order to predict the target phosphorylation sites from the sequence- and structure-based analysis of the specific protein kinases' catalytic domain, such as KinasePhos2.0 [46] or GPS [47]. In particular, GPS is a group-based phosphorylation algorithm, which predicts kinase-specific phosphorylation sites in 71 protein kinase groups, such as Aurora-A, Aurora-B and NimA-like protein kinases. Other methods were instead implemented in a way to predict the phosphorylation sites simply from the substrate primary sequences. For example, Scansite [48] is built on combined experimental binding and/or substrate information to derive a weighted matrix-based scoring that predicts protein-protein and protein-phospholipid interactions, as well as phosphorylation sites. NetPhos [49] instead is based on an artificial neuronal network that allows the users to choose between a generic predictions based only on the substrate protein sequence or kinase-specific predictions.

Recently, in order to overcome the limitations due to a training set based only on the same type of kinases, two general predictors were developed: PPRED [50], which incorporates evolutionary information, and PhosphOrtholog [51] that enables cross-species comparison of large-scale phosphorylation sites. Finally, several online databases are also available in order to curate and organize information about phosphorylation sites studied *in vivo* and *in vitro* in human and mouse proteomes (PhosphositePlus [52]), as well as rat, fly, yeast and worm (PhosphoELM [53]).

### 2.2 Glycosylation

Protein glycosylation is one of the most relevant and complex post-translational modifications in the cell [54], [55], which is thought to influence almost half of all proteins in nature [56]. It consists of a covalent interaction between a glycosyl donor of a glycan and a glycosyl acceptor amino acid side chain of a protein [57] (Fig. 1B). Protein glycosylation can be divided in four main categories based on the linkage between the amino acid and sugar: N-linked glycans, O-linked glycans, GPI anchors and C-mannosylation. In N-glycosylation, a sugar is attached to an amino group of an asparagine [58], while O-glycosylation is characterized by the interaction of a sugar with the hydroxyl group of a serine or threonine [59]. GPI anchors consist of the attachment of glycophosphatidyl-inositol near to the C-terminal of a protein chain anchoring the protein to the membrane [60]. C-mannosylation occurs when an α-mannopyranosyl moiety is attached to the indole of the tryptophan via C—C link [61].

Glycosylation modulates several protein biophysical properties influencing their native functions [2]. In particular, it was observed that it could alter not only protein thermodynamic and kinetic properties, but also influence the structural features of the proteins [62]. The covalent attachment of large hydrophilic carbohydrates modulates protein stability, oligomerization and aggregation [62], [63], [64], host cell-surface interactions [65], enzyme activity [66] and protein trafficking [67] (Table 2).

Several analytical tools were developed over the past 2–3 decades facilitating glycan analysis. In particular, capillary electrophoresis, liquid chromatography, mass spectrometry and microarray-based are extensively used in grycoproteomics [68], [69], [70]. None of these tools can produce, however, a detailed molecular characterization of glycosylated proteins. The high heterogeneity of glycans and the difficulty of obtaining them in large amounts still preclude investigating the role of glycosylation at the molecular level. In the past years, the number of glycoconjugates' crystallographic protein structures have increased [71], [72], [73], [74], [75], [76], nevertheless, a complete chemical and structural description of a glycan structure is still challenging. Mass spectrometry as well as different web-servers (Table 1) currently provide information about existing glycosylation sites. Indeed, in the last decade, several algorithms, trained with sequences or sequence-based information, have been developed to improve prediction of glycosylation sites. Some of these resources are based on neuronal network algorithms, such as NetNGlyc, NetOGlyc [77] for prokaryotes, or YingOYang [78] for eukaryotes. Other useful tools are the GlycoMod [79] server for prediction of glycans' structure based on experimental determined masses, and the NGlyPred [80] server, which incorporates both structure and residue pattern information. More recent developments include prediction of glycosylation sites based on machine learning algorithms (*i.e.*, GlycoMine [81]), an approach that has produced a significant improvement with respect to prediction performances of NetNGlyc [82] and NectOGlyc [77].

### 2.3 S-nitrosylation

S-nitrosylation (SNO) consists in the covalent attachment of a nitric oxide (NO) to cysteine thiol moieties (Fig. 1C). Compared to phosphorylation, SNO is not catalyzed by an enzyme, but it depends on the chemical reactivity between the nitrosylation agent and the target, thus the specific residues' environment influences the reactivity of the target protein. Concentration of the nitrosylation agent and the protein, as well as the stability of the S—NO bond under physiological conditions, influences in turn the specificity of this reaction.

Over the past 2 decades, hundreds of soluble [83], [84], [85], [86], [87], [88], [89], [90], [91] or membrane [92], [93] proteins have been identified to be S-nitrosylated. The SNO modification not only modulates protein stability and activities [94], [95], but also plays an important role in a variety of biological processes, such as cell signaling, transcriptional regulation, apoptosis and chromatin remodeling [96] (Table 2). Increasing evidences indicate that aberrant S-nitrosylation is implicated in various diseases like cancer [97], Parkinson's [98], [99], Alzheimer's [100] and amyotrophic lateral sclerosis [101]. Thus the identification of SNO sites in proteins can be also very important for the development of drugs.

Although S—NO bonds are highly labile and redox-sensitive, several techniques managed to detect SNO in cells. There are methods for the direct detection of S-nitrosylated sites, such as the measurement of S—NO characteristic absorbance at 340 nm, electrospray ionization mass spectrometry (ESI-MS) [102] and NMR with ^15^N [103]. Ozone chemiluminescence [104] and specific reduction with Cu^+^/cysteine [105] at pH 6 are indirect chemical methods that are instead based on the analysis of the cleavage products of SNO. Biotin switch assays and chemical reduction/chemiluminescence assays are specific and sensitive methods for measuring low levels of intracellular S-nitrosylated proteins. These experiments are laborious and low-throughput due to the labile nature and low abundance of SNO. Therefore, computational methods represent again a valid alternative to timely and reliably identifying SNO protein sites for further experimental verification. Several benchmark datasets were developed during the past years. SNOSID [106] tests the prediction performance on 65 positive and 65 negative samples, while GPS-NO [107] was developed based on 549 experimentally verified SNO sites. A support vector algorithm machine (SVM) [108] and a nearest neighbor algorithm (NNA) [109] were also proposed to predict SNO sites. However, no web server was later developed for any of these methods, so that their current usage is quite limited. Alternative web-servers are iSNO-PseAAc [110], which identifies nitrosylated proteins on an independent dataset, predicting sites with 90% accuracy [110], and GPS-SNO [107], which also represents a valid tool for an experimentalist providing information for hundreds of potentially S-nitrosylated substrates that have not been yet experimentally determined [110] (Table 1).

### 2.4 Methylation

Protein methylation is a reversible PTM that modifies the nitrogen atoms of either the backbone or side-chain of several types of amino acids, such as lysine, arginine, histidine, alanine and asparagine [111], [112], [113], [114], [115], [116], [117]; methylation has been also reported at cysteine residues (S-methylation) [118] (Fig. 1D). Despite this variability, most studies have been predominantly focused on lysine and arginine modifications. Methylation research dates back to 1939, but just recently has attracted more and more attention [111] with the identification of new methyltransferases, like protein arginine methyltransferases (PRMTs) [119], [120], [121] or histone lysine methyltransferases (HKMTs) [122], [123], [124], which catalyze mono [125] or double [111], [126] methylation. In particular, the methylation of the N-terminal tails of the histone plays an important role in gene expression regulation [127], genome stability [128] and nuclear architecture [129] influencing several biological processes such as transcription [130], [131] and chromosome maintenance [132] (Table 2). Methylation can also occur on the C-5 position of the cytosine ring of the DNA (DNA methylation) resulting in its association with several human diseases such as cancer, mental retardation (Angelman syndrome) or diabetes mellitus [133]. Although different biological processes are linked to DNA and histone methylation, there seems to be a mutual relationship between these processes, which could play an important role in gene expression [134].

Methylated proteins, as well as methylation regulatory enzymes, are involved in several human diseases such as cancer [126], [135], [136], cardiovascular diseases [137], multiple sclerosis [138] and neurodegenerative disorders [139]. Thus, the inhibition of these enzymes with small molecules could be an effective therapeutic means of intervention [140]. Moreover, as it is key to identifying methylation sites, understanding methylation mechanistic and dynamic features is as important. In the past years several experimental methods were developed to study the molecular mechanism of methylation. Mutagenesis of potential methylated residues, methylation of a specific antibody [141], as well as Chip-Chip [142] were extensively used for this purpose. Recently, mass spectrometry experiments have been also applied allowing the identification of 249 arginine methylated protein sites in 131 proteins from T cells [143]. However, these techniques are usually very expensive and laborious limiting the research of potential methylation sites.

Computational predictions of methylation sites have helped handle these limitations providing an important resource for reducing the number of experiments needed to determine protein methylation sites. Eight web-servers for prediction of methylation sites are currently available (Table 1). MeMo [144] is one of the first online tools to become available. It uses a support vector machine (SVM) as a prediction algorithm. Its dataset is based on a curated selection of all methylated residues annotated in SWISS-PROT [145], 264 experimentally manually verified methyl-lysine and 107 methyl-arginine extracted from roughly 1700 scientific articles. MeMo [144] appears to be a powerful tool for predicting methylated-arginine sites when compared to methylated-lysine. However, its accuracy is affected by the lack of training data available at the time of development. Lately, the reliability of the prediction was improved by BPB-PPMS [146], where a Bi-Profile Bayesian approach was used to define methylated and non-methylated sites based on known experimental data [147], [148]. The data set was increased to 363 candidates containing methylated arginines and 977 methylated lysine proteins. The combination of Bi-Profile Bayesian features with a larger data set improved the methylation prediction accuracy up to 92% for methylated lysine proteins and 88% for methylated arginine proteins [149]. It was observed that protein methylation mainly occurs in regions that are easily accessible and intrinsically disordered, thus MASA [149] used Solvent Accessible Surface Area (SASA) and secondary structure information for predicting methylated sites. This web-server allows the prediction not only of methylated lysines and methylated arginines, but also methyl-glutamates. However, most of these methods use only primary sequence information without taking into account any physicochemical property of residues. With the aim of improving the quality of the prediction, a novel approach called PMes [150] was introduced, which considers physiochemical properties of amino acids surrounding methylation sites. A specific lysine-methylation prediction tool for histones was also proposed: METhK [151] uses amino acids' composition, SASA, amino acid pair composition (*i.e.*, the frequency of amino acid pairs in the primary sequence), amino acid index and protein disorder regions for discriminating between methylated lysine sites in histones and in non-histone proteins. More recently, another web-server has been introduced for *in vivo* or *in vitro* species-specific methylation sites' identification: PSSMe [152] was tested on a large-scale experimental methylated site dataset originated from different species, revealing that methylation patterns are indeed species dependent.

### 2.5 N-acetylation

Protein acetylation is a covalent post-translational modification where the acetyl group from acetyl coenzyme A (acetyl CoA) is transferred either to the α-amino group of terminal residues (N^α^-acetylation) or to the ε-amino group of internal lysine at specific sites (N^ε^-acetylation) [153], [154], [155], [156], [157] (Fig. 1E). Although N^α^-acetylation is more common (roughly 85% in eukaryotic proteins), N^ε^-lysine acetylation is more biologically important [156], [157], [158], [159], [160], [161], [162], [163]. Indeed N^ε^-acetylation on internal lysines is a reversible post-translational modification involved in several biological processes, such as transcription regulation [159], [161], protein expression and stability [153], [164], [165], [166], [167], DNA repair [162], apoptosis [160], [163] and nuclear import [158] (Table 2). Aberrant lysine acetylation is linked with cancer [157], [168], [169], [170], neurodegenerative disorders [171], [172], [173] and cardiovascular diseases [174], [175], [176], [177], [178]. Thus, the identification of acetylation sites is important for shedding light on the acetylation mechanism at the basis of numerous diseases [179].

Experimentally several techniques were applied to explore N-acetylation, such as radioactivity detection [180], immunity affinity detection and chromatin immunoprecipitation [181]. The development of high-throughput technologies like immune-precipitation combined to mass spectrometry increased also the number of detected acetylated proteins [182]. However, the experimental detection of acetylated sites is inefficient, expensive and have implicitly low throughput [183]. Therefore, computational tools represent alternative methods for studying the acetylation modifications and provide information for further experiments. Some web-servers (Table 1) dealt only with one specific type of acetylation such as NetAcet [184] for instance. NetAcet [184] attempted to predict only N^α^-acetylation sites using a neuronal network trained on yeast data and extendable only to mammalian acetylated substrates. However, NetAcet [184] suffered from the limited size of the training dataset available at that time of development. Several web-servers aimed to predict acetylated lysine. PAIL [185] was the first *in silico* tool for N^ε^-lysine acetylation sites' prediction. The Bayesian discriminant algorithm [186] was employed on a training set of 246 experimentally verified acetylated sites. Despite a small data set, PAIL [185] is able to achieved an accuracy of 85%. BRABSB-PHKA [187] is a human-specific lysine acetylation predictor, which combines a bi-relative adaptive binomial score Bayesian algorithm with a support vector machine. Another method in lysine acetylation prediction is PSKace-Pred [188], where a position-specific view was considered for the characterization of acetylated proteins.

Protein sequences' information, evolution similarity and physiochemical properties can help in discriminating between acetyl-lysines and non-acetyl-lysines, improving lysine sites' evaluation. LAceP [189] is based on a logistic regression model, where the physiochemical property of the amino acids and the transition probability of adjacent amino acids were considered during the prediction process. It also allows predicting acetylated sites not only for lysines, but also for glycine, methionine serine and threonine residues. This is actually done by N-Ace [190], where physiochemical properties (*e.g.*, non-bonded energy, absolute entropy) and solvent accessibility were included in the original prediction code.

The status of lysine acetylation can also be influenced by the enzymes that catalyze the reaction. Although lysine acetyltransferases (KATs) act usually on a multiple-subunit complex, it is still difficult to determine which KATs are responsible for the acetylation of a given protein. ASEB [191] was the first server for KAT-specific human acetylated lysine prediction that not only evaluates possible lysine acetylation sites, but also provides information about the responsible KAT enzyme.

## 3 Covalent attachment of acyl chains

Protein lipidation is a unique post-translational modification, which has the result of directly controlling the interaction of soluble protein with biological membranes affecting in turn cellular organization and trafficking. In this section we give an overview of several types of lipidation, their mechanism, involvement in diseases and the computational resources used for predicting lipidation sites.

### 3.1 Palmitoylation

Palmitoylation consists in the attachment of a 16-carbon acyl chain to cysteine residues via a thioesteric bond [192], [193] (Fig. 1F). Among all PTM lipidations, palmitoylation is the only reversible one and can dynamically regulate protein function, as in the case of H-Ras and N-Ras [3], [194]. Two families of enzymes regulate the palmitoylation/depalmitoylation process: palmitoyltransferases (PATs), which catalyze the attachment of a palmitate from CoA to specific cysteines, and Acyl Protein Thioesterases (APTs), which remove the palmitate acyl chain. Palmitoylation occurs both in soluble and membrane proteins playing a critical role in the regulation of key biological processes, such as protein membrane trafficking, signaling, cell growth and development [195] (Table 2). Aberrant palmitoylation is associated to a variety of human diseases including neurological disorders (*e.g.*, Huntington disease's [196] or Alzheimer's disease [197]) and cancer [198], [199], [200], [201], [202], [203]. However, the S-palmitoylated proteome is not yet well defined and little is known about the mechanism that regulates S-palmitoylation and its consequences. In fact, the identification of palmitoylation sites is not simple due to the lack of a distinct sequence motif on the substrates [204]. Mass spectrometry allows the identification of several palmitoylated proteins in cells and tissues, which can be further experimentally characterized using Acyl Biotin Exchange (ABE) or Acyl Resin Assisted Capture (Acyl-RAC) techniques [205], [206], [207]. Metabolic labeling and click chemistry probes [208], [209] were developed to recognize palmitoylation sites in order to shed light on the molecular mechanism and dynamics of palmitoylation. All these experimental techniques are time and money consuming, thus computer-aided methods are a necessary alternative for predicting palmitoylation sites (Table 1). CSS-palm [210] was one of the first methods to be developed for searching novel palmitoylated proteins in budding yeast. It is based on a clustering and scoring algorithm, where 263 experimentally verified palmitoylation sites are used as a training set, manually collected from the scientific literature. CKSAAP-PALM [211] is another computational method to predict palmitoylation sites based on protein sequences. An encoding scheme composed by *k*-spaced amino acid pairs is at the basis of this approach [211], which improved accuracy compared to former strategies. SwissPalm [212] has been recently introduced, which provides information from the comparison of different palmitoyl-proteomic studies and allows the users to easily search for the protein of interest, determine the predicted S-palmitoylation sites, identify orthologues and compare them across palmitoyl-proteomes. SeqPalm [213] has been recently developed in order to get insights into the correlation between the disruption of palmitoylation sites and diseases. This new computational method allows for the identification of palmitoylation sites based on amino acid compositions, autocorrelation of amino acid physicochemical properties and amino acid position-weighted matrices.

### 3.2 N-myristoylation

Myristoylation is a covalent and irreversible attachment of a 14-carbon fatty acid to N-terminal Gly residues [214] of eukaryotic or viral proteins (Fig. 1G). This PTM facilitates in turn the interaction with membranes or a hydrophobic protein domain [215], [216], [217], [218], [219]. The substrates involved in myristoylation are generally characterized by the consensus motif Met-Gly-X-X-X-Ser/Thr at the N-terminus. This PTM acts predominantly by removal of the main methionine residues in order to expose the subsequent glycine [220]. Less frequently, it can also expose an internal glycine by proteases' cleavage [221]. These mechanisms are both catalyzed by the N-myristoyl transferase (NMT), a 50 kDa enzyme expressed in most organisms [222], [223].

Myristoylation is involved in several critical cellular processes, such as signaling pathways, apoptosis [221] and extracellular protein export [224] (Table 2). Usually myristoylation acts with other post-translational modifications like palmitoylation [225], [226], [227], or in combination with positively charged residues [228] in order to enhance membrane-protein interactions. Several diseases are linked to N-myristoylation like cancer, epilepsy, Alzheimer's disease and viral and bacterial infections [229]. The experimental detection of N-myristoylation includes radioactive techniques like the use of ^3^H or ^14^C radioactive myristate that requires a long exposure period (weeks to months). To the best of our knowledge only two online web-servers are available to predict myristoylation sites (Table 1). NMT [230] uses a trial set that combines experimentally proved myristoylated proteins with potential myristoylated candidates. Based on structural and biochemical characterization of the N-myristoyl-transferase, a set of descriptors was suggested for better predicting myristoylated sites. This protocol improved the previous pattern suggested in PROSITE [231] (pattern code: PDOC00008), which gave numerous false negative predictions.

Another N-myristoylated site predictor is called Myristoylator [232], which is based on a machine learning model that uses several combined neuronal networks and a test set of positive and negative sequences. Although this predictor seems to increase the specificity, it was trained to predict myristoylation only on terminal glycines, thus a priori knowledge of the proteolytic scission site is necessary when using this web-server.

### 3.3 Prenylation

Prenylation is a PTM leading to the attachment of a 15-carbon (farnesylation) or a 20-carbon (geranylgeranylation) lipid to cysteines catalyzed by farnesyltransferases or by protein geranlygeranyl transferases I, respectively (Fig. 1H). These isoprenyl anchors promote not only protein-membrane [233], [234], [235], [236], [237], but also protein-protein interactions [238], [239], [240] (Table 2). Several diseases are correlated to this PTM, like cancer [241], [242], premature aging disorders [243], [244], neurite [244] and hepatites C and D [245]. Protein prenylation occurs also in a wide range of parasites, leading to the use of protein farnesyltransferase inhibitors in protozoan parasitic diseases [246].

The most common approach for detecting prenylation is to use expensive radiolabeling techniques [203], [204]. Initially, the prenylation motif was suggested to be CaaX, *i.e.* consisting of a cysteine (C) followed by two aliphatic residues (aa) and a terminal residue X. However, further kinetic studies and mutation experiments showed a more flexible and complex recognition motif for prenylation [247]. PrePS [248] is the only online tool available, which is based on modeling of the substrate-enzyme interactions for each prenyltransferase.

## 4 Small proteins' modifications

An important field in cell signaling is the characterization of the covalent and reversible attachment of ubiquitin (ubiquitylation) and small ubiquitin-related modifiers (sumoylation). This peculiar class of PTMs provides new protein-protein interfaces remodeling the target proteins [249]. In this section we review the latest findings on sumoylation and ubiquitylation with a particular attention on the *in silico* tools recently developed.

### 4.1 Ubiquitylation

Ubiquitylation is a three step process where, first, the ubiquitin is activated by a ubiquitin-activating enzyme (E1), then conjugated to a ubiquitin-conjugating enzyme (E2), and finally transferred by a ubiquitin-ligase enzyme (E3) to a substrate molecule via an isopeptide bond with an internal lysine (Fig. 1I). This reversible modification is implicated in the regulation of several cellular processes, like protein degradation [250], [251], [252], cell cycle division, the immune response [253], lysosomal trafficking [254] and control of insulin [255] (Table 2). The aberration of ubiquitylation is linked to human pathologies varying from inflammatory neurodegenerative diseases to different forms of cancers [253], [256].

Despite the availability of several ubiquitin-protein ligase complex structures [257], [258], [259], [260], [261], the ubiquitylation reaction mechanism is still poorly understood. It has been recently hypothesized that structural disorders of the substrate could actually facilitate this process. Analysis of sequences by mutant yeast strain experiments [262] showed that most of the ubiquitylation sites are in the disordered and flexible regions of a protein. On the basis of this observation UbPred was developed [262] (Table 1): a ubiquitylation site predictor based on a support vector machine algorithm (SVM), which allows studying the correlation between ubiquitylation and protein half-life. In order to overcome the lack of accuracy and training data deficiency, UbiProber [263] and iUbiq [264] were designed (Table 1). UbiProber predicts both general and species-specific ubiquitylation sites using large-scale experimental data as training set, while iUbiq is based on evolutionary information incorporated into the general form of pseudo-amino acid composition. However, all these *in silico* tools do not account for E3 binding/recognition sites, although it was shown to be an important feature for ubiquitylation. UbiNet [265] (Table 1) is the first server that allows studying the regulatory network among E3 and ubiquitylated proteins.

### 4.2 Sumoylation

Sumoylation is a PTM characterized by a covalent attachment of the Small Ubiquitin-like Modifier (SUMO) to specific lysine residues via an enzymatic reaction (Fig. 1L). Sumoylation sites are identified by a canonical consensus sequence Ψ-K-X-E (where Ψ is a hydrophobic amino acid, such as A, I, L, M, P, F, V or W; X any amino acid residue) [266], [267], and by SUMO-interacting motifs (3–4 aliphatic residues linked to acid and/or phosphorylatable amino acids) called SIM [268]. Both these features are essential for characterizing the biological significance of sumoylation. This modification is involved in several cellular processes, like protein binding, subcellular transport, gene expression, DNA repair, chromosome assembly and cellular signaling [269], [270], [271], [272] (Table 2). Aberrant sumoylation is correlated not only to Alzheimer's and Parkinson's diseases [273], but also to cancer [274] and diabetes [275], highlighting the importance of detecting sumoylation sites. Mass spectrometry-based proteomic studies allow mapping hundreds of proteins identifying different sumoylation and SIM sites [276], [277], [278], [279]. However, the limitations due to the reversibility of this modification and the difficult identification of peptides from trypsin digestion impose some limitations to the use of this technique. Computer-aided prediction represents a good alternative to reduce the number of potential targets to explore for further experimental verifications (Table 1).

While web-servers available for SIM prediction are only GPS-SUMO [280] and JASSA [281], several online methods for sumoylation sites' prediction are currently available. SUMOhydro [282] is based on a support vector machine (SVM) combined with amino acid hydrophobicity, while SUMOAMVR [283] considers also other structural features, like average accessible surface area (AASA), secondary structure and evolutionary information of amino acids. Recently, a new *in silico* tool based on the covariance discriminant (CD) algorithm was developed in order to avoid errors caused by disparity in training data sets [284].

## 5 PTMs cross-talk

The hypothesis of a correlation between PTMs within the same protein (PTMs cross-talk) [285] has emerged in the proteomic field in recent years. For instance, the regulatory interplay among PTMs was observed for histones [286] and other proteins like p53 [287], [288], RNA polymerase II [288] or β-tubulin [289]. In particular, the importance of PTM cross-talk was recognized in several biological pathways (*e.g.* DNA damage response [290] and protein stability regulation [291], [292], [293]) pointing to a strong relationship between PTM cross-talk and protein functions.

While the structural and functional understanding of combinatorial PTMs has been initially limited by technological limitations, recent advances in proteomics have allowed integrating information on different types of modifications [294], [295]. However, with the latest experimental methods it is also difficult to identify the whole set of PTM sites in the proteins. In this emerging context, computational methods are poised to support the study of PTM cross-talk. The first unified tool for a simultaneous prediction of PTM sites was ModPred [296], which predicts and analyses simultaneously multiple types of PTM sites in order to gain structural and functional information on the protein regulatory mechanism of multiple PTMs. Recently, a new webserver, PTM-X [297], allows the prediction of PTM cross-talk based on experimentally published data. The difference compared to ModPred is represented by the necessity to know a priori the PTM candidate sites.

## 6 Structural and dynamical characterization of PTMs

Despite the important role played by PTMs, their structural and dynamics effects of protein function remain poorly understood from a molecular point of view, due to the labile transient nature of most of these modifications and the lack of adequate experimental techniques able to detect and characterize them and the underlying chemical mechanism of formation. The online tools previously discussed are valid methods to overcome some of these experimental limitations and predict putative PTMed sites, but they do not usually provide any information about the impact of post-translational modifications from a mechanistic point of view.

Molecular modeling and molecular simulation (such as molecular dynamics, MD), which are based on empirical atomistic force fields [298], [299], [300], [301], are a powerful strategy for studying biological systems at single molecule resolution and nanosecond-to-millisecond time scales. Although this computational approach allows nowadays the study of protein processes and properties that are not easily accessible experimentally, there are still some apparent limitations regarding the availability of accurate parameters that would allow the investigation of PTMed proteins. In the past years several improvements have been made in order to expand this approach also to non-standard biomolecules. Within the AMBER force field atomic charges and parameters were developed for phosphorylated residues as phosphoserine, phosphothreonine, phosphotyrosine, phosphohistidine [302], and S-nitrosylated residues (S-nitrocysteine [303]) and methylation (trimethyllysine [304], [305]). Similarly, within the CHARMM force field there are parameters for methylated lysines and arginines, as well as acetylated lysines and palmitoylated cysteines [306]. There are also *ad hoc* comprehensive atomistic force field parameters for treating the description of the link between carbohydrates and proteins such as in GLYCAM for AMBER [307]/CHARMM [308] and a modified version of GROMOS [309], [310]. In theory, within these schemes, there are existing strategies to parameterize virtually any kind of non-standard amino acids, as for the case of PTMs; in practice, the development of new force field models always involves time-consuming parameterization protocols and rigorous a posteriori validations of the quality and robustness of the new models.

Moving to lower resolution, coarse-grained force fields can be also very useful for studying the impact of PTMs on protein function. In this domain there are no specific parameters for the description of PTMed residues. The Martini force field [311], for instance, provides parameters for treating non-covalently bound sugar molecules or phosphate groups but a complete general representation of modified residues is not yet available. However, a recent work described new parameters for modeling palmitoylated cysteines [312] that were used to study H-Ras, and contributed to show the influence of this PTM in regulating the partition of the protein with the membrane.

While for a long time PTMs were not usually considered in modeling and molecular simulation works, the recent availability of more comprehensive experimental data along with accurate force field parameters have thus allowed investigating protein properties taking also into account the effect of PTMs on their structure and stability. Recent examples of this approach have revealed the impact of PTMs for the HIV-1 fusion peptide structure [313], rhodopsin [314], calnexyn [315] and phosphatidylinositol 4-kinase [316].

Answering to the need of new and better molecular models to more realistically describe proteins, some automatic tools for generating force field parameters for new molecular species have become available, such as ParaChem or SwissParam [317] compatible with the CHARMM force field, q4md-forcefieldtools for AMBER [318] and ATB for GROMOS [319]. However, none of them directly focus on the parameterization of PTMs, likely because of the complexity of the development of parameters required for most PTMs. Therefore, the necessity of having computational tools allowing an automatic parameterization of PTMed protein structures to be used in MD simulations resulted in the development of some new web-servers, such as FF_PTM (http://selene.princeton.edu/FFPTM/) and Vienna-PTM (http://vienna-ptm.univie.ac.at). FF_PTM focuses on expanding the existing AMBER force field (*i.e.*, ff03) including parameters for 32 PTMs. In particular, it is characterized by parameters that describe the attachment of small molecules (*e.g.*, phosphorylation, methylation or acetylation) and the covalent interaction with acyl chains such as palmitic acid (palmitoylation) and geranylgeranyl pyrophosphate (geranylgeranylation). On the other hand, Vienna-PTM is a web platform designed for introducing PTMs on PDB structures to run simulations using the GROMOS 54A7 and 45A3 force fields.

## 7 Summary and outlook

The chemical modification of amino acids plays an important role in a myriad of cellular processes that range from protein localization to disease development and aging. Enormous efforts, which combine the development of both experimental and computational methods, have been put in the past 2 decades in order to understand PTM mechanisms and effects for protein structure, dynamics and function.

In this review we summarized the main *in silico* tools mainly available as an online webserver for studying PTMs (Table 1). Recently, an integrative platform (dbPTM: http://dbptm.mbc.nctu.edu.tw/) has also become available. Originally developed as a comprehensive database of experimentally verified PTMs, dbPTM collects all available databases and webserver resources considering also other PTMs, like succinylation and S-glutathionylation. Although this platform does not provide an exhaustive description for the case of lipidation, it includes also standalone software (not discussed here), offering thus a complementary source of information to this review.

With an increasing amount of experimental data available every day, we think that, as the existing ones will keep improving their performance, many other methods will emerge in the future. Although most of the web-servers available are based on a sequence-based analysis of training data sets, some of them have also started to take into account other interesting properties, such as evolutionary information (*e.g.*, PhosphoOrtholog, iUbiq), SASA (*e.g.*, MASA, METhK, SUMOAMVR) and physiochemical properties (*e.g.*, Pmes, SUMOhydro).

Altogether, these approaches are only rarely considering the molecular features associated with PTMs and the molecular impact they have for protein function in general. Within this context, molecular modeling and simulations have the capability to complement experimental and bioinformatics techniques providing a molecular description of the effect of PTMs on protein structures and stability. However, the lack of suitable tools and parameters for treating PTMs in proteins has limited so far the characterization of these covalent modifications. As some automatic tools (*e.g.*, FF_PTM and Vienna-PTM) have recently appeared providing an online platform to parameterize post-translational modified proteins suitable for running atomistic MD simulations with AMBER or GROMOS force fields, for most PTMs *ad hoc* parameterizations still need to be developed. Similarly, coarse-grained force fields still lack reliable and robust models for dealing with PTMs, as well as systematic protocols to produce accurate parameters.

Nowadays, with the constant increment of computing power and the availability of always more accurate force fields for biomolecules, which accompany the tireless advances on the experimental side, it is possible to achieve a more precise and faithful description of biological systems in their physiological conditions using molecular modeling and simulation. For instance, the advances in lipidomic analyses have provided a much more detailed view of membrane composition, allowing the construction of more realistic membrane models [320], [321] to better investigate membrane biophysical properties and their interplay with integral and peripheral membrane proteins [322], [323]. Several computational tools have been developed with this aim in mind, such as CHARMM-GUI [308] and LipidBuilder [324].

Along the same lines, it is also clear that protein PTMs are another important layer of complexity that integrally defines and modulates protein function and, for this reason, needs to be considered at all levels of both experimental and computational investigation. Therefore, the computational advances of bioinformatics and physics-based molecular modeling and simulation techniques appear as a fundamental requirement to complement the experimental investigation of PTMs' impact on cellular processes.

## Figures and Tables

**Table 1:** PTM prediction webservers. Abbreviations: artificial neuronal network (ANN); support vector machine (SVM); random forest method (RFM); Hidden Markov model (HMM); weight matrix (WM); group based phosphorylation scoring method (GPS); binary profile of patterns (BPP); composition profile of patterns (CPP); PSSM profile of patterns (PPM); average surface accessibility (ASA); neuronal network (NN); knowledge-based (KB); conditional random field (CRF); group-based prediction (GBP); binary profile bayesian (BPB); information gain (IG); Bayesian discriminant (BD); enrichment based method (EBM); binary-relative adaptive binomial score Bayesian (Bi-BSP); logistic regression model (LRM); synthetic minority oversampling technique (SMOT); Markov chain clustering (MCC); particle swarm optimization (PSO); genetic variability (GV); position frequency matrix (PFM); covariance discriminant algorithm (CD): machine learning (ML).

PTM type		Covalent attachment of small chemical groups Web server and URL	Year	Description	Method	Information
Phosphorylation	NetPhos 3.1	http://www.cbs.dtu.dk/services/NetPhos/	1999	K-specific and K-independent	ANN	Prediction based on 17 different kinases
	Scansite	http://scansite.mit.edu	2003	K-specific	WM	Identification of short protein sequence motifs that are recognized by modular signaling domains or mediated specific interaction with proteins
	PhosphoSitePlus	http://www.phosphosite.org/siteSearchAction.action	2004	K-specific	–	Repository of human and mouse phosphorylation sites
	GPS	http://gps.biocuckoo.org/online.php	2005	K-specific	GPS	Prediction based on 71 PK groups (*e.g.* Aurora-A, Aurora-B and NIMA)
	KinasePhos 2.0	http://kinasephos2.mbc.nctu.edu.tw	2007	K-specific	SVM	SVM coupled with protein coupling pattern
	PhosphoELM	http://phospho.elm.eu.org	2010	K-independent	–	Repository of *in vivo* and *in vitro* phosphorylation site*s*
	PPRED	http://biomecis.uta.edu/~ashis/res/ppred/	2010	K-independent	SVM	Prediction based on evolutionary information
	PhosPhortholog	http://www.phosphortholog.com	2015	K-independent	–	Database for cross-species comparison
Glycosylation	bigPI	http://mendel.imp.ac.at/gpi/gpi_server.html	1999	GPI-anchor	KB	Prediction for protozoa and metazoa
	O-GlycBase	http://www.cbs.dtu.dk/databases/OGLYCBASE/	1999	O-glycosylated	–	Repository of O-glycosylated proteins based on protein sequence database and scientific literature
	GlycoMod	http://web.expasy.org/glycomod/	2001	N-,O-glycosylated	Experimental determined	Match between the experimentally determined masses and the predicted protease (SWISSPROT and TrEMBL databases)
	YinOYang	http://www.cbs.dtu.dk/services/YinOYang/	2001	N-,C-,O-glycosylated	NN	Prediction based on eukaryotes protein sequences
	NetNGlyC	http://www.cbs.dtu.dk/services/NetNGlyc/	2002	N-glycosylated	NN	Prediction for procaryotes
	GlyProt	http://www.glycosciences.de/glyprot/	2005	N-glycosylated	SWEET-II	3D model of glycoproteins based on a PDB structure without attached glycans
	GPP	http://comp.chem.nottingham.ac.uk/glyco/	2008	N-,C-,O-glycosylated	RF	Prediction of glycosylation sites and the propensity of association with modified residues
	NGlycPred	https://exon.niaid.nih.gov/nglycpred/	2012	N-glycosylated	RF	Combination of different structure and residues pattern information
	GLYCOPP	http://www.imtech.res.in/raghava/glycopp/submit.html	2012	N-,O-glycosylated	SVM	Prediction based on different approaches (BPP, CPP, PPP, ASA + BPP)
	NetOGlyC	http://www.cbs.dtu.dk/services/NetOGlyc/	2013	O-glycosylated	NN	Prediction for prokaryotes
S-nitrosylation	GlycoMine	http://www.structbioinfor.org/Lab/GlycoMine/#webserver	2015	N-,C-,O-glycosylated	RF	Determination of the features important for glycosylation site specificity
	GPS-SNO	http://sno.biocuckoo.org/online.php	2010	SNO sites	GBP	Prediction of putative SNO based on a database of 504 experimentally verified SNO
Methylation	iSNO-PseAAC	http://app.aporc.org/iSNO-PseAAC/	2013	SNO sites	CRF	Identification of nitrosylated protein on an independent data set (731 experimentally verified SNO and 810 experimentally non verified SNO)
	MeMo	http://www.bioinfo.tsinghua.edu.cn/~tigerchen/memo.html	2006	R-,L-methylated	SVM	Prediction based on orthogonal binary coding scheme for representing protein sequence fragments
	BPB-PPMS	http://www.bioinfo.bio.cuhk.edu.hk/bpbppms/	2009	R-,L-methylated	BPB and SVM	Prediction based on experimental data
	MASA	http://masa.mbc.nctu.edu.tw/	2009	K-,R-,E-,N-methylated	SVM	Prediction based on structural information (SASA and secondary structures)
	PMes	http://bioinfo.ncu.edu.cn/inquiries_PMeS.aspx	2012	R-,K-methylated	SVM	Prediction based on physiochemical properties (VdW volume, position weight aminoacid, composition, solvent, SASA)
	MethK	http://csb.cse.yzu.edu.tw/MethK/	2014	K-methylated histone	SVM	Differentiation between K-methylated Histone and K-methylated non-Histone
	iMethyl-PseAAC	http://www.jci-bioinfo.cn/iMethyl-PseAAC	2014	R-,K-methylated	SVM	Prediction based on physiochemical properties, sequence evolution, biochemical and structural disorder information
N-acetylation	PSSMe	http://bioinfo.ncu.edu.cn/PSSMe.aspx	2016	R-,L-methylated	IGF	Prediction based on species-specific models
	NetAcet	http://www.cbs.dtu.dk/services/NetAcet/	2004	Nα-acetylated	NN	Prediction for yeast and mammalian
	PAIL	http://bdmpail.biocuckoo.org/prediction.php	2006	Nε-,K-acetylated	BD	Prediction based on dataset of 246 acetylated substrates
	N-Ace	http://n-ace.mbc.nctu.edu.tw	2010	K-,A-,G-,M-,S- and T-acetylated	SVM	Prediction based on physiochemical properties
	ASEB	http://bioinfo.bjmu.edu.cn/huac/	2012	K-acetylated	EBM	Prediction based on protein-protein interaction information
	BRABSB-PHKA	http://www.bioinfo.bio.cuhk.edu.hk/bpbphka/	2012	K-acetylated	Bi-BSB	Prediction for human-specific lysine acetylated sites
	PSKacePred	http://bioinfo.ncu.edu.cn/inquiries_PSKAcePred.aspx	2012	K-acetylated	SVM	Prediction based on amynoacid composition, evolutionary similarity and physiochemical properties
	LAceP	http://www.scbit.org/iPTM/	2014	K-acetylated	LRM	Prediction based on physiochemical properties


PTM type		Covalent attachment of acyl chains Web server and URL	Year	Description	Method	Information
Palmitoylation	CSS-Palm	http://csspalm.biocuckoo.org/online.php	2008	Palmitoylated sites	Clustering and scoring algorithm	Prediction for budding yeast
	CKSAAP-Palm	http://doc.aporc.org/wiki/CKSAAP-Palm	2009	Palmitoylated sites	SVM	Prediction based on protein sequences
	SwissPalm	http://swisspalm.epfl.ch	2015	Palmitoylated sites	–	Repository of different palmitoylation-proteomic studies
N-myristoylation	SeqPalm	http://lishuyan.lzu.edu.cn/seqpalm/	2015	Palmitoylated sites	SMOT	Correlation between the disruption of palmitoylation sites and diseases
	NMT	http://mendel.imp.ac.at/myristate/SUPLpredictor.htm	2002	N-myristoylated sites	PSIC algorithm	Identification of the N-myristoylated sites processing terminal glycine or internal glycine
Prenylation	Myristoylator	http://web.expasy.org/myristoylator/	2004	N-myristoylated sites	NN	Supplementary tool for NMT.
	PrePS	http://mendel.imp.ac.at/sat/PrePS/	2005	Farnesylated and geranygeranylated	MCC	Prediction based on first sequences and physical properties


PTM type		Small proteins Web server and URL	Year	Description	Method	Information
Sumoylation	SUMOhydro	http://protein.cau.edu.cn/others/SUMOhydro/	2012	Sumoylated sites	SVM	Prediction based on amino acid hydrophobicity
	GPS-SUMO	http://sumosp.biocuckoo.org/online.php	2014	Sumoylated sites	GPS and PSO	Investigation on the relationship between sumoylation and sumo interaction process
	SUMOAMVR	http://bioinfo.ncu.edu.cn/SUMOAMVR_Prediction.aspx	2014	Sumoylated sites	GV	Investigation on the impact of sumo sites in human diseases
	JASSA	http://www.jassa.fr	2015	Sumoylated sites	PFM	Identification of database hits, analysis of physicochemical properties and systematic pattern search
	pSumo-CD	http://www.jci-bioinfo.cn/pSumo-CD	2016	Sumoylated sites	CD	Prediction based on the integration of sequence-coupled information into general pseudo-aminoacid composition
Ubiquitylation	UbPred	http://www.ubpred.org	2009	Ubiquitylated sites	RF	Prediction based on first sequences and structural information
	UbiProber	http://bioinfo.ncu.edu.cn/ubiprober.aspx	2013	Ubiquitylated sites	ML	Prediction of species-specific ubiquitinated sites from experimental data
	iUbiq-Lys	http://www.jci-bioinfo.cn/iUbiq-Lys	2014	Ubiquitylated sites	RF	Prediction based on evolutionary information
	UbiNet	http://140.138.144.145/~ubinet/index.php	2016	E3 binding/recognition sites	-	Repository of experimental data, ubiquitylated substrates and protein-protein interactions

**Table 2:**
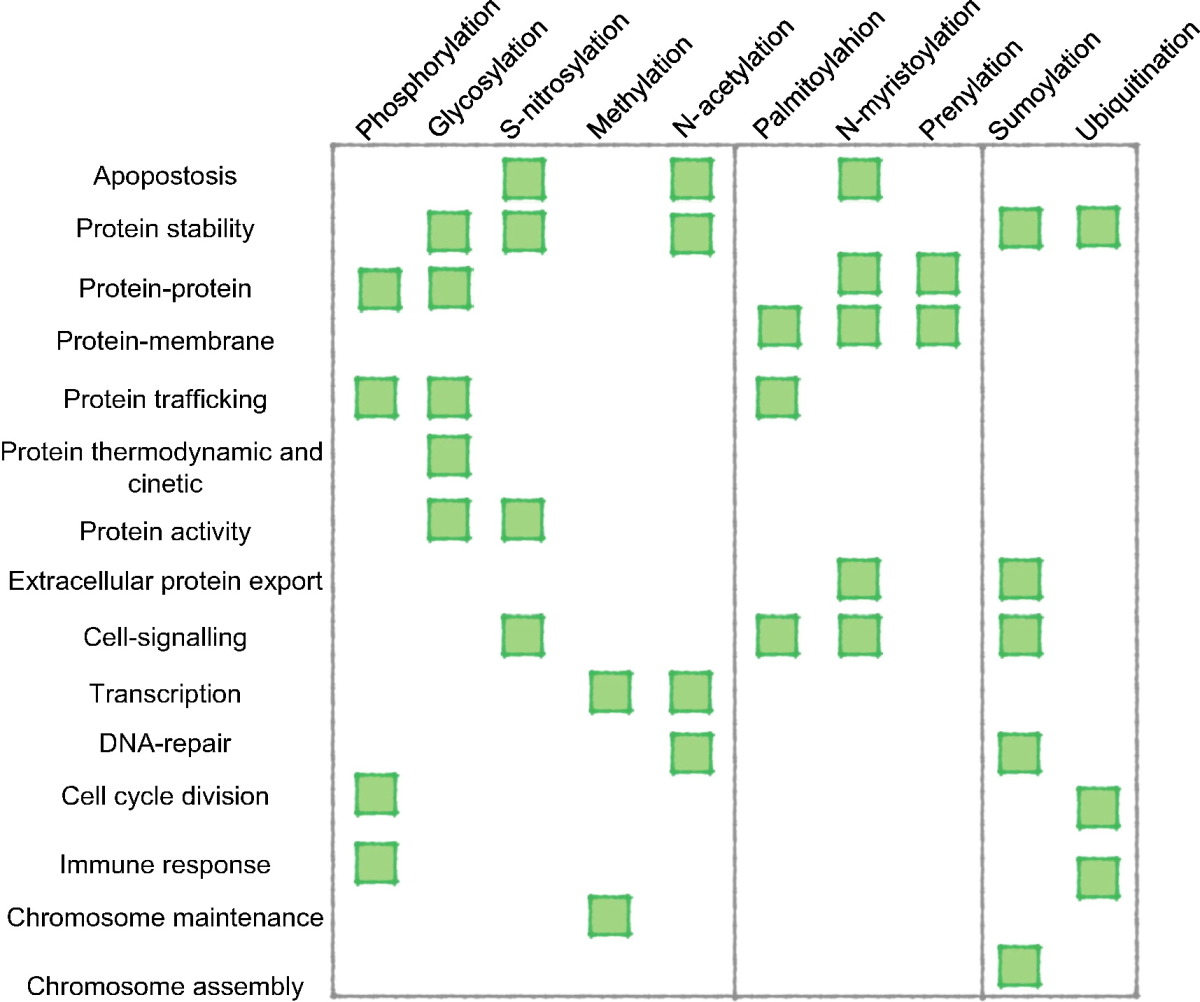
Schematic relationship between PTMs and their implication in biological functions.

## References

[1] Walsh C. (2006). Posttranslational modification of proteins: Expanding nature's inventory.

[2] Walsh C.T. (2005). Protein posttranslational modifications: the chemistry of proteome diversifications. Angew Chem Int Ed.

[3] Rocks O. (2005). An acylation cycle regulates localization and activity of palmitoylated Ras isoforms. Science.

[4] Fairbank M. (2012). RING finger palmitoylation of the endoplasmic reticulum Gp78 E3 ubiquitin ligase. FEBS Lett.

[5] Maeda A. (2010). Palmitoylation stabilizes unliganded rod opsin. Proc Natl Acad Sci.

[6] Hunter T. (2009). Tyrosine phosphorylation: thirty years and counting. Curr Opin Cell Biol.

[7] Khoury G.A. (2011). Proteome-wide post-translational modification statistics: frequency analysis and curation of the swiss-prot database. Sci Rep.

[8] Venne A.S. (2015). An improved workflow for quantitative N-terminal charge-based fractional diagonal chromatography (ChaFRADIC) to study proteolytic events in *Arabidopsis thaliana*. Proteomics.

[9] Premont R. (1995). Protein kinases that phosphorylate activated G protein-coupled receptors. FASEB J.

[10] Gurevich E.V. (2012). G protein-coupled receptor kinases: more than just kinases and not only for GPCRs. Pharmacol Ther.

[11] Gao C. (2014). Regulation of Wnt/β-catenin signaling by posttranslational modifications. Cell Biosci.

[12] Jiang W. (2013). Histone H3K27me3 demethylases KDM6A and KDM6B modulate definitive endoderm differentiation from human ESCs by regulating WNT signaling pathway. Cell Res.

[13] Ren R.-J. (2014). Proteomics of protein post-translational modifications implicated in neurodegeneration. Transl Neurodegener.

[14] Krueger K.E. (2006). Posttranslational protein modifications current implications for cancer detection, prevention, and therapeutics. Mol Cell Proteomics.

[15] McLaughlin R.J. (2016). Where, how, and when: positioning posttranslational modification within type 1 diabetes pathogenesis. Curr Diab Rep.

[16] Aebersold R. (2003). Mass spectrometry-based proteomics. Nature.

[17] Lothrop A.P. (2013). Deciphering post-translational modification codes. FEBS Lett.

[18] Zhang J. (2012). PEAKS DB: de novo sequencing assisted database search for sensitive and accurate peptide identification. Mol Cell Proteomics.

[19] He L. (2014). GlycoMaster DB: software to assist the automated identification of N-linked glycopeptides by tandem mass spectrometry. J Proteome Res.

[20] Na S. (2012). Fast multi-blind modification search through tandem mass spectrometry. Mol Cell Proteomics.

[21] Cohen P. (2002). The origins of protein phosphorylation. Nat Cell Biol.

[22] Mandell D.J. (2007). Strengths of hydrogen bonds involving phosphorylated amino acid side chains. J Am Chem Soc.

[23] Manning G. (2002). The protein kinase complement of the human genome. Science.

[24] Manning G. (2002). Evolution of protein kinase signaling from yeast to man. Trends Biochem Sci.

[25] Johnson L.N. (2001). Structural basis for control by phosphorylation. Chem Rev.

[26] Johnson L. (1993). The effects of phosphorylation on the structure and function of proteins. Annu Rev Biophys Biomol Struct.

[27] Russo A.A. (1996). Structural basis of cyclin-dependent kinase activation by phosphorylation. Nat Struct Mol Biol.

[28] Hurley J. (1990). Regulation of isocitrate dehydrogenase by phosphorylation involves no long-range conformational change in the free enzyme. J Biol Chem.

[29] Welburn J.P. (2007). How tyrosine 15 phosphorylation inhibits the activity of cyclin-dependent kinase 2-cyclin A. J Biol Chem.

[30] Filippakopoulos P. (2008). Structural coupling of SH2-kinase domains links Fes and Abl substrate recognition and kinase activation. Cell.

[31] Macdonald N. (2005). Molecular basis for the recognition of phosphorylated and phosphoacetylated histone h3 by 14-3-3. Mol Cell.

[32] Lowery D.M. (2005). Structure and function of Polo-like kinases. Oncogene.

[33] Ferrarese A. (2007). Chemical dissection of the APC Repeat 3 multistep phosphorylation by the concerted action of protein kinases CK1 and GSK3. Biochemistry.

[34] Canagarajah B.J. (1997). Activation mechanism of the MAP kinase ERK2 by dual phosphorylation. Cell.

[35] Becker S. (1998). Three-dimensional structure of the Stat3β homodimer bound to DNA. Nature.

[36] Chen X. (1998). Crystal structure of a tyrosine phosphorylated STAT-1 dimer bound to DNA. Cell.

[37] Rubin S.M. (2005). Structure of the Rb C-terminal domain bound to E2F1-DP1: a mechanism for phosphorylation-induced E2F release. Cell.

[38] Antz C. (1999). Control of K^+^ channel gating by protein phosphorylation: structural switches of the inactivation gate. Nat Struct Mol Biol.

[39] Vénien-Bryan C. (2009). The structure of phosphorylase kinase holoenzyme at 9.9 Å resolution and location of the catalytic subunit and the substrate glycogen phosphorylase. Structure.

[40] Pinna L.A. (1996). How do protein kinases recognize their substrates?. Biochimi Biophy Acta Mol Cell Res.

[41] Cohen P. (2000). The regulation of protein function by multisite phosphorylation—a 25 year update. Trends Biochem Sci.

[42] Humphrey S.J. (2013). Dynamic adipocyte phosphoproteome reveals that Akt directly regulates mTORC2. Cell Metab.

[43] Huttlin E.L. (2010). A tissue-specific atlas of mouse protein phosphorylation and expression. Cell.

[44] Olsen J.V. (2010). Quantitative phosphoproteomics reveals widespread full phosphorylation site occupancy during mitosis. Sci Signal.

[45] Sharma K. (2014). Ultradeep human phosphoproteome reveals a distinct regulatory nature of Tyr and Ser/Thr-based signaling. Cell Rep.

[46] Wong Y.-H. (2007). KinasePhos 2.0: a web server for identifying protein kinase-specific phosphorylation sites based on sequences and coupling patterns. Nucleic Acids Res.

[47] Xue Y. (2008). GPS 2.0, a tool to predict kinase-specific phosphorylation sites in hierarchy. Mol Cell Proteomics.

[48] Obenauer J.C. (2003). Scansite 2.0: proteome-wide prediction of cell signaling interactions using short sequence motifs. Nucleic Acids Res.

[49] Blom N. (2004). Prediction of post-translational glycosylation and phosphorylation of proteins from the amino acid sequence. Proteomics.

[50] Biswas A.K. (2010). Machine learning approach to predict protein phosphorylation sites by incorporating evolutionary information. BMC Bioinf.

[51] Chaudhuri R. (2015). PhosphOrtholog: a web-based tool for cross-species mapping of orthologous protein post-translational modifications. BMC Genomics.

[52] Hornbeck P.V. (2004). PhosphoSite: a bioinformatics resource dedicated to physiological protein phosphorylation. Proteomics.

[53] Dinkel H. (2011). Phospho. ELM: a database of phosphorylation sites—update 2011. Nucleic Acids Res.

[54] Freeze H.H. (2009). Other Classes of ER/Golgi-derived Glycans.

[55] Schwarz F. (2011). Mechanisms and principles of N-linked protein glycosylation. Curr Opin Struct Biol.

[56] Apweiler R. (1999). On the frequency of protein glycosylation, as deduced from analysis of the SWISS-PROT database. Biochim Biophys Acta Gen Subj.

[57] Spiro R.G. (1973). Glycoproteins. Adv Protein Chem.

[58] Imperiali B. (1995). Asparagine-linked glycosylation: specificity and function of oligosaccharyl transferase. Bioorg Med Chem.

[59] Jentoft N. (1990). Why are proteins O-glycosylated?. Trends Biochem Sci.

[60] Eisenhaber B. (1999). Prediction of potential GPI-modification sites in proprotein sequences. J Mol Biol.

[61] Hofsteenge J. (1994). New type of linkage between a carbohydrate and a protein: C-glycosylation of a specific tryptophan residue in human RNase Us. Biochemistry.

[62] Mitra N. (2006). N-linked oligosaccharides as outfitters for glycoprotein folding, form and function. Trends Biochem Sci.

[63] Hanson S.R. (2009). The core trisaccharide of an N-linked glycoprotein intrinsically accelerates folding and enhances stability. Proc Natl Acad Sci.

[64] Bosques C.J. (2003). The interplay of glycosylation and disulfide formation influences fibrillization in a prion protein fragment. Proc Natl Acad Sci.

[65] Imberty A. (2008). Microbial recognition of human cell surface glycoconjugates. Curr Opin Struct Biol.

[66] Skropeta D. (2009). The effect of individual N-glycans on enzyme activity. Bioorg Med Chem.

[67] Lederkremer G.Z. (2009). Glycoprotein folding, quality control and ER-associated degradation. Curr Opin Struct Biol.

[68] Steentoft C. (2013). Glycoengineering of human cell lines using zinc finger nuclease gene targeting: SimpleCells with homogeneous GalNAc O-glycosylation allow isolation of the O-glycoproteome by one-step lectin affinity chromatography. Glycosyltransferases.

[69] Steentoft C. (2011). Mining the O-glycoproteome using zinc-finger nuclease-glycoengineered SimpleCell lines. Nat Methods.

[70] Mechref Y. (2011). Analysis of glycans derived from glycoconjugates by capillary electrophoresis-mass spectrometry. Electrophoresis.

[71] Deisenhofer J. (1981). Crystallographic refinement and atomic models of a human Fc fragment and its complex with fragment B of protein A from *Staphylococcus aureus* at 2.9- and 2.8-Å resolution. Biochemistry.

[72] Idusogie E.E. (2000). Mapping of the C1q binding site on rituxan, a chimeric antibody with a human IgG1 Fc. J Immunol.

[73] Krapp S. (2003). Structural analysis of human IgG-Fc glycoforms reveals a correlation between glycosylation and structural integrity. J Mol Biol.

[74] Oganesyan V. (2008). Structural characterization of a mutated, ADCC-enhanced human Fc fragment. Mol Immunol.

[75] Mizushima T. (2011). Structural basis for improved efficacy of therapeutic antibodies on defucosylation of their Fc glycans. Genes Cells.

[76] Ferrara C. (2011). Unique carbohydrate–carbohydrate interactions are required for high affinity binding between FcγRIII and antibodies lacking core fucose. Proc Natl Acad Sci.

[77] Steentoft C. (2013). Precision mapping of the human O-GalNAc glycoproteome through SimpleCell technology. EMBO J.

[78] Gupta R. (2001). Prediction of glycosylation across the human proteome and the correlation to protein function. Pac Symp Biocomput.

[79] Cooper C.A. (2001). GlycoMod—a software tool for determining glycosylation compositions from mass spectrometric data. Proteomics.

[80] Chuang G.-Y. (2012). Computational prediction of N-linked glycosylation incorporating structural properties and patterns. Bioinformatics.

[81] Li F. (2015). GlycoMine: a machine learning-based approach for predicting N-, C-and O-linked glycosylation in the human proteome. Bioinformatics.

[82] Gupta R. (2004). Prediction of N-glycosylation Sites in Human Proteins.

[83] Choi Y.-B. (2000). Molecular basis of NMDA receptor-coupled ion channel modulation by S-nitrosylation. Nat Neurosci.

[84] Schonhoff C.M. (2002). Nitric oxide-mediated inhibition of Hdm2-p53 binding. Biochemistry.

[85] Park H.-S. (2000). Nitric oxide negatively regulates c-Jun N-terminal kinase/stress-activated protein kinase by means of S-nitrosylation. Proc Natl Acad Sci.

[86] Matsushita K. (2003). Nitric oxide regulates exocytosis by S-nitrosylation of N-ethylmaleimide-sensitive factor. Cell.

[87] Mannick J.B. (2001). S-nitrosylation of mitochondrial caspases. J Cell Biol.

[88] Mannick J.B. (1999). Fas-induced caspase denitrosylation. Science.

[89] Lander H.M. (1997). A molecular redox switch on p21ras structural basis for the nitric oxide-p21ras interaction. J Biol Chem.

[90] Haendeler J. (2002). Redox regulatory and anti-apoptotic functions of thioredoxin depend on S-nitrosylation at cysteine 69. Nat Cell Biol.

[91] Li S. (2003). Regulation of protein tyrosine phosphatase 1B in intact cells by S-nitrosothiols. Arch Biochem Biophys.

[92] Broillet M.-C. (2000). A single intracellular cysteine residue is responsible for the activation of the olfactory cyclic nucleotide-gated channel by NO. J Biol Chem.

[93] Eu J.P. (2000). The skeletal muscle calcium release channel: coupled O_2_ sensor and NO signaling functions. Cell.

[94] Derakhshan B. (2007). Unbiased identification of cysteine S-nitrosylation sites on proteins. Nat Protoc.

[95] Tsang A.H. (2009). S-nitrosylation of XIAP compromises neuronal survival in Parkinson's disease. Proc Natl Acad Sci.

[96] Nott A. (2008). S-nitrosylation of histone deacetylase 2 induces chromatin remodelling in neurons. Nature.

[97] Aranda E. (2012). Nitric oxide and cancer: the emerging role of S-nitrosylation. Curr Mol Med.

[98] Yao D. (2004). Nitrosative stress linked to sporadic Parkinson's disease: S-nitrosylation of parkin regulates its E3 ubiquitin ligase activity. Proc Natl Acad Sci U S A.

[99] Uehara T. (2006). S-nitrosylated protein-disulphide isomerase links protein misfolding to neurodegeneration. Nature.

[100] Cho D.-H. (2009). S-nitrosylation of Drp1 mediates β-amyloid-related mitochondrial fission and neuronal injury. Science.

[101] Schonhoff C.M. (2006). S-nitrosothiol depletion in amyotrophic lateral sclerosis. Proc Natl Acad Sci U S A.

[102] Chen Y.-Y. (2007). Mass spectrometry-based analyses for identifying and characterizing S-nitrosylation of protein tyrosine phosphatases. Methods.

[103] Živković M.L. (2012). Post-translational S-nitrosylation is an endogenous factor fine tuning the properties of human S100A1 protein. J Biol Chem.

[104] Martínez-Ruiz A. (2005). S-nitrosylation of Hsp90 promotes the inhibition of its ATPase and endothelial nitric oxide synthase regulatory activities. Proc Natl Acad Sci U S A.

[105] Fang K. (1998). Reductive assays for S-nitrosothiols: implications for measurements in biological systems. Biochem Biophys Res Commun.

[106] Hao G. (2006). SNOSID, a proteomic method for identification of cysteine S-nitrosylation sites in complex protein mixtures. Proc Natl Acad Sci U S A.

[107] Xue Y. (2010). GPS-SNO: computational prediction of protein S-nitrosylation sites with a modified GPS algorithm. PLoS One.

[108] Li Y.-X. (2011). An efficient support vector machine approach for identifying protein S-nitrosylation sites. Protein Pept Lett.

[109] Li B.-Q. (2012). Predict and analyze S-nitrosylation modification sites with the mRMR and IFS approaches. J Proteomics.

[110] Xu Y. (2013). iSNO-PseAAC: predict cysteine S-nitrosylation sites in proteins by incorporating position specific amino acid propensity into pseudo amino acid composition. PLoS One.

[111] Bannister A.J. (2005). Reversing histone methylation. Nature.

[112] Bedford M.T. (2005). Arginine methylation: an emerging regulator of protein function. Mol Cell.

[113] Boisvert F.-M. (2005). Protein interfaces in signaling regulated by arginine methylation. Sci STKE.

[114] Cheng X. (2005). Structural and sequence motifs of protein (histone) methylation enzymes. Annu Rev Biophys Biomol Struct.

[115] Fackelmayer F.O. (2005). Protein arginine methyltransferases: guardians of the Arg?. Trends Biochem Sci.

[116] Lee D.Y. (2005). Role of protein methylation in regulation of transcription. Endocr Rev.

[117] Martin C. (2005). The diverse functions of histone lysine methylation. Nat Rev Mol Cell Biol.

[118] Lapko V.N. (2005). Modifications of human βA1/βA3-crystallins include S-methylation, glutathiolation, and truncation. Protein Sci.

[119] Xie B. (2007). Arginine methylation of the human immunodeficiency virus type 1 Tat protein by PRMT6 negatively affects Tat interactions with both cyclin T1 and the Tat transactivation region. J Virol.

[120] Cheng D. (2007). The arginine methyltransferase CARM1 regulates the coupling of transcription and mRNA processing. Mol Cell.

[121] Torres-Padilla M.-E. (2007). Histone arginine methylation regulates pluripotency in the early mouse embryo. Nature.

[122] Barski A. (2007). High-resolution profiling of histone methylations in the human genome. Cell.

[123] Porras-Yakushi T.R. (2007). Yeast ribosomal/cytochrome c SET domain methyltransferase subfamily identification of Rpl23ab methylation sites and recognition motifs. J Biol Chem.

[124] Shi X. (2007). Modulation of p53 function by SET8-mediated methylation at lysine 382. Mol Cell.

[125] Pahlich S. (2006). Protein arginine methylation: cellular functions and methods of analysis. Biochim Biophys Acta Proteins Proteomics.

[126] Varier R.A. (2011). Histone lysine methylation and demethylation pathways in cancer. Biochim Biophys Acta Rev Cancer.

[127] Shi Y. (2004). Histone demethylation mediated by the nuclear amine oxidase homolog LSD1. Cell.

[128] Peters A.H. (2001). Loss of the Suv39h histone methyltransferases impairs mammalian heterochromatin and genome stability. Cell.

[129] Black J.C. (2011). Chromatin landscape: methylation beyond transcription. Epigenetics.

[130] Mikkelsen T.S. (2007). Genome-wide maps of chromatin state in pluripotent and lineage-committed cells. Nature.

[131] Santos-Rosa H. (2002). Active genes are tri-methylated at K4 of histone H3. Nature.

[132] Black J.C. (2012). Histone lysine methylation dynamics: establishment, regulation, and biological impact. Mol Cell.

[133] Robertson K.D. (2005). DNA methylation and human disease. Nat Rev Genet.

[134] Cedar H. (2009). Linking DNA methylation and histone modification: patterns and paradigms. Nat Rev Genet.

[135] Aletta J.M. (1998). Protein methylation: a signal event in post-translational modification. Trends Biochem Sci.

[136] Paik W.K. (2007). Historical review: the field of protein methylation. Trends Biochem Sci.

[137] Chen X. (2006). Expression of nitric oxide related enzymes in coronary heart disease. Basic Res Cardiol.

[138] Mastronardi F.G. (2006). Increased citrullination of histone H3 in multiple sclerosis brain and animal models of demyelination: a role for tumor necrosis factor-induced peptidylarginine deiminase 4 translocation. J Neurosci.

[139] Longo V.D. (2006). Sirtuins in aging and age-related disease. Cell.

[140] Copeland R.A. (2009). Protein methyltransferases as a target class for drug discovery. Nat Rev Drug Discov.

[141] Boisvert F.-M. (2003). A proteomic analysis of arginine-methylated protein complexes. Mol Cell Proteomics.

[142] Johnson David S. (2008). "Systematic evaluation of variability in ChIP-chip experiments using predefined DNA targets.". Genome research.

[143] Snijders A.P. (2010). Analysis of arginine and lysine methylation utilizing peptide separations at neutral pH and electron transfer dissociation mass spectrometry. J Am Soc Mass Spectrom.

[144] Chen H. (2006). MeMo: a web tool for prediction of protein methylation modifications. Nucleic Acids Res.

[145] Boeckmann B. (2003). The SWISS-PROT protein knowledgebase and its supplement TrEMBL in 2003. Nucleic Acids Res.

[146] Shao J. (2009). Computational identification of protein methylation sites through bi-profile Bayes feature extraction. PLoS One.

[147] Grant J.E. (2007). Post-translational modifications in the rat lumbar spinal cord in experimental autoimmune encephalomyelitis. J Proteome Res.

[148] Xie B. (2003). Replication of human immunodeficiency viruses engineered with heterologous Tat-transactivation response element interactions. J Virol.

[149] Shien D.M. (2009). Incorporating structural characteristics for identification of protein methylation sites. J Comput Chem.

[150] Shi S.-P. (2012). PMeS: prediction of methylation sites based on enhanced feature encoding scheme. PLoS One.

[151] Lee T.-Y. (2014). Identification and characterization of lysine-methylated sites on histones and non-histone proteins. Comput Biol Chem.

[152] Wen P.-P. (2016). Accurate in silico prediction of species-specific methylation sites based on information gain feature optimization. Bioinformatics.

[153] Glozak M.A. (2005). Acetylation and deacetylation of non-histone proteins. Gene.

[154] Kouzarides T. (2000). Acetylation: a regulatory modification to rival phosphorylation?. EMBO J.

[155] Polevoda B. (2000). Nα-terminal acetylation of eukaryotic proteins. J Biol Chem.

[156] Polevoda B. (2002). The diversity of acetylated proteins. Genome Biol.

[157] Yang X.J. (2004). The diverse superfamily of lysine acetyltransferases and their roles in leukemia and other diseases. Nucleic Acids Res.

[158] Bannister A.J. (2000). Acetylation of importin-α nuclear import factors by CBP/p300. Curr Biol.

[159] Brunet A. (2004). Stress-dependent regulation of FOXO transcription factors by the SIRT1 deacetylase. Science.

[160] Cohen H.Y. (2004). Acetylation of the C terminus of Ku70 by CBP and PCAF controls Bax-mediated apoptosis. Mol Cell.

[161] Faiola F. (2005). Dual regulation of c-Myc by p300 via acetylation-dependent control of Myc protein turnover and coactivation of Myc-induced transcription. Mol Cell Biol.

[162] Murr R. (2006). Histone acetylation by Trrap–Tip60 modulates loading of repair proteins and repair of DNA double-strand breaks. Nat Cell Biol.

[163] Subramanian C. (2005). Ku70 acetylation mediates neuroblastoma cell death induced by histone deacetylase inhibitors. Proc Natl Acad Sci U S A.

[164] Kamita M. (2011). N α-acetylation of yeast ribosomal proteins and its effect on protein synthesis. J Proteomics.

[165] Kurdistani S.K. (2003). Histone acetylation and deacetylation in yeast. Nat Rev Mol Cell Biol.

[166] Kuo M.-L. (2004). N-terminal polyubiquitination and degradation of the Arf tumor suppressor. Genes Dev.

[167] Behnia R. (2004). Targeting of the Arf-like GTPase Arl3p to the Golgi requires N-terminal acetylation and the membrane protein Sys1p. Nat Cell Biol.

[168] Kikuno N. (2008). Genistein mediated histone acetylation and demethylation activates tumor suppressor genes in prostate cancer cells. Int J Cancer.

[169] Yang X. (2007). HATs and HDACs: from structure, function and regulation to novel strategies for therapy and prevention. Oncogene.

[170] Geng H. (2011). HDAC4 protein regulates HIF1α protein lysine acetylation and cancer cell response to hypoxia. J Biol Chem.

[171] Mihm M.J. (2007). Cardiac dysfunction in the R6/2 mouse model of Huntington's disease. Neurobiol Dis.

[172] Chen K.-C. (2012). OxLDL causes both epigenetic modification and signaling regulation on the microRNA-29b gene: novel mechanisms for cardiovascular diseases. J Mol Cell Cardiol.

[173] Iyer A. (2012). Lysine acetylation in obesity, diabetes and metabolic disease. Immunol Cell Biol.

[174] Jeong H. (2009). Acetylation targets mutant huntingtin to autophagosomes for degradation. Cell.

[175] Dompierre J.P. (2007). Histone deacetylase 6 inhibition compensates for the transport deficit in Huntington's disease by increasing tubulin acetylation. J Neurosci.

[176] Ko M.H. (2009). Two endoplasmic reticulum (ER)/ER Golgi intermediate compartment-based lysine acetyltransferases post-translationally regulate BACE1 levels. J Biol Chem.

[177] Jonas M.C. (2008). PCSK9 is required for the disposal of non-acetylated intermediates of the nascent membrane protein BACE1. EMBO Rep.

[178] Cong X. (2011). Mass spectrometric identification of novel lysine acetylation sites in huntingtin. Mol Cell Proteomics.

[179] Mottet D. (2008). Histone deacetylases: target enzymes for cancer therapy. Clin Exp Metastasis.

[180] Welsch D.J. (1988). Amino-terminal alanine functions in a calcium-specific process essential for membrane binding by prothrombin fragment 1. Biochemistry.

[181] Umlauf D. (2004). Site-specific Analysis of Histone Methylation and Acetylation. Epigenetics Protocols.

[182] Choudhary C. (2009). Lysine acetylation targets protein complexes and co-regulates major cellular functions. Science.

[183] Basu A. (2009). Proteome-wide prediction of acetylation substrates. Proc Natl Acad Sci.

[184] Kiemer L. (2005). NetAcet: prediction of N-terminal acetylation sites. Bioinformatics.

[185] Li A. (2006). Prediction of N ε-acetylation on internal lysines implemented in Bayesian Discriminant Method. Biochem Biophys Res Commun.

[186] Xue Y. (2006). PPSP: prediction of PK-specific phosphorylation site with Bayesian decision theory. BMC Bioinf.

[187] Shao J. (2012). Systematic analysis of human lysine acetylation proteins and accurate prediction of human lysine acetylation through bi-relative adapted binomial score Bayes feature representation. Mol Biosyst.

[188] Suo S.-B. (2012). Position-specific analysis and prediction for protein lysine acetylation based on multiple features. PLoS One.

[189] Hou T. (2014). LAceP: lysine acetylation site prediction using logistic regression classifiers. PLoS One.

[190] Lee T.Y. (2010). N-Ace: using solvent accessibility and physicochemical properties to identify protein N-acetylation sites. J Comput Chem.

[191] Wang L. (2012). ASEB: a web server for KAT-specific acetylation site prediction. Nucleic Acids Res.

[192] Salaun C. (2010). The intracellular dynamic of protein palmitoylation. J Cell Biol.

[193] Blaskovic S. (2013). What does S-palmitoylation do to membrane proteins?. FEBS J.

[194] Lynch S.J. (2015). The differential palmitoylation states of N-Ras and H-Ras determine their distinct Golgi subcompartment localizations. J Cell Physiol.

[195] Martin B.R. (2012). Global profiling of dynamic protein palmitoylation. Nat Methods.

[196] Singaraja R.R. (2011). Altered palmitoylation and neuropathological deficits in mice lacking HIP14. Hum Mol Genet.

[197] Mizumaru C. (2009). Suppression of APP-containing vesicle trafficking and production of u-amyloid by AID/DHHC-12 protein. J Neurochem.

[198] Iwabuchi M. (2014). Characterization of the ubiquitin-modified proteome regulated by transient forebrain ischemia. J Cereb Blood Flow Metab.

[199] Yamamoto Y. (2007). Gain of 5p15. 33 is associated with progression of bladder cancer. Oncology.

[200] Kang R. (2008). Neural palmitoyl-proteomics reveals dynamic synaptic palmitoylation. Nature.

[201] Oyama T. (2000). Isolation of a novel gene on 8p21. 3–22 whose expression is reduced significantly in human colorectal cancers with liver metastasis. Genes Chromosomes Cancer.

[202] Birkenkamp-Demtroder K. (2002). Gene expression in colorectal cancer. Cancer Res.

[203] Yeste-Velasco M. (2014). Identification of ZDHHC14 as a novel human tumour suppressor gene. J Pathol.

[204] Munday A.D. (2007). Posttranslational protein palmitoylation promoting platelet purpose. Arterioscler Thromb Vasc Biol.

[205] Roth A.F. (2006). Global analysis of protein palmitoylation in yeast. Cell.

[206] Wan J. (2007). Palmitoylated proteins: purification and identification. Nat Protoc.

[207] Forrester M.T. (2011). Site-specific analysis of protein S-acylation by resin-assisted capture. J Lipid Res.

[208] Martin B.R. (2009). Large-scale profiling of protein palmitoylation in mammalian cells. Nat Methods.

[209] Tsai F.D. (2014). Metabolic Labeling of Ras with Tritiated Palmitate to Monitor Palmitoylation and Depalmitoylation. Ras Signaling: Methods and Protocols.

[210] Ren J. (2008). CSS-Palm 2.0: an updated software for palmitoylation sites prediction. Protein Eng Des Sel.

[211] Wang X.-B. (2009). Prediction of palmitoylation sites using the composition of k-spaced amino acid pairs. Protein Eng Des Sel.

[212] Blanc M. (2015). SwissPalm: protein palmitoylation database. F1000Res.

[213] Li S. (2015). In silico identification of protein S-palmitoylation sites and their involvement in human inherited disease. J Chem Inf Model.

[214] Carr S.A. (1982). N-tetradecanoyl is the NH_2_-terminal blocking group of the catalytic subunit of cyclic AMP-dependent protein kinase from bovine cardiac muscle. Proc Natl Acad Sci.

[215] Towler D. (1988). The biology and enzymology of eukaryotic protein acylation. Annu Rev Biochem.

[216] Gordon J.I. (1990). Protein N-myristoylation: simple questions, unexpected answers. Clin Res.

[217] Kia-Ki H. (1992). Post-translational chemical modification(s) of proteins. Int J Biochem.

[218] Johnson D.R. (1994). Genetic and biochemical studies of protein N-myristoylation. Annu Rev Biochem.

[219] Farazi T.A. (2001). Structures of *Saccharomyces cerevisiae* N-myristoyltransferase with bound myristoylCoA and peptide provide insights about substrate recognition and catalysis. Biochemistry.

[220] Wilcox C. (1987). Acylation of proteins with myristic acid occurs cotranslationally. Science.

[221] Zha J. (2000). Posttranslational N-myristoylation of BID as a molecular switch for targeting mitochondria and apoptosis. Science.

[222] Giang D.K. (1998). A second mammalian N-myristoyltransferase. J Biol Chem.

[223] Glover C.J. (1995). Identification and characterization of multiple forms of bovine brain N-myristoyltransferase. J Biol Chem.

[224] Denny P.W. (2000). Acylation-dependent protein export inLeishmania. J Biol Chem.

[225] Mill P. (2009). Palmitoylation regulates epidermal homeostasis and hair follicle differentiation. PLoS Genet.

[226] Tsutsumi R. (2009). Identification of G protein α subunit-palmitoylating enzyme. Mol Cell Biol.

[227] Fukata M. (2004). Identification of PSD-95 palmitoylating enzymes. Neuron.

[228] McLaughlin S. (1995). The myristoyl-electrostatic switch: a modulator of reversible protein-membrane interactions. Trends Biochem Sci.

[229] Thinon E. (2014). Global profiling of co-and post-translationally N-myristoylated proteomes in human cells. Nat Commun.

[230] Maurer-Stroh S. (2002). N-terminal N-myristoylation of proteins: prediction of substrate proteins from amino acid sequence. J Mol Biol.

[231] Sigrist C.J. (2010). PROSITE, a protein domain database for functional characterization and annotation. Nucleic Acids Res.

[232] Bologna G. (2004). N-terminal myristoylation predictions by ensembles of neural networks. Proteomics.

[233] Pillinger M.H. (1994). Characterization of a plasma membrane-associated prenylcysteine-directed alpha carboxyl methyltransferase in human neutrophils. J Biol Chem.

[234] Manolaridis I. (2013). Mechanism of farnesylated CAAX protein processing by the intramembrane protease Rce1. Nature.

[235] Yang J. (2011). Mechanism of isoprenylcysteine carboxyl methylation from the crystal structure of the integral membrane methyltransferase ICMT. Mol Cell.

[236] Diver M.M. (2014). Mutational analysis of the integral membrane methyltransferase isoprenylcysteine carboxyl methyltransferase (ICMT) reveals potential substrate binding sites. J Biol Chem.

[237] Bhagatji P. (2010). Multiple cellular proteins modulate the dynamics of K-ras association with the plasma membrane. Biophys J.

[238] Hoffman G.R. (2000). Structure of the Rho family GTP-binding protein Cdc42 in complex with the multifunctional regulator RhoGDI. Cell.

[239] Jaffe A.B. (2005). Rho GTPases: biochemistry and biology. Annu Rev Cell Dev Biol.

[240] Nishimura A. (2013). Identification of a novel prenyl and palmitoyl modification at the CaaX motif of Cdc42 that regulates RhoGDI binding. Mol Cell Biol.

[241] Barbacid M. (1987). Ras genes. Annu Rev Biochem.

[242] Khosravi-Far R. (1994). The Ras signal transduction pathway. Cancer Metastasis Rev.

[243] Amoyel M. (2013). Isoprenylcysteine carboxylmethyltransferase deficiency exacerbates KRAS-driven pancreatic neoplasia via Notch suppression. J Clin Invest.

[244] Winter-Vann A.M. (2005). A small-molecule inhibitor of isoprenylcysteine carboxyl methyltransferase with antitumor activity in cancer cells. Proc Natl Acad Sci U S A.

[245] Ye J. (2003). Disruption of hepatitis C virus RNA replication through inhibition of host protein geranylgeranylation. Proc Natl Acad Sci.

[246] VWC Van (2006). Fighting parasitic disease by blocking protein farnesylation. J Lipid Res.

[247] Casey P.J. (1996). Protein prenyltransferases. J Biol Chem.

[248] Maurer-Stroh S. (2005). Refinement and prediction of protein prenylation motifs. Genome Biol.

[249] Denuc A. (2009). The UBA-UIM domains of the USP25 regulate the enzyme ubiquitination state and modulate substrate recognition. PLoS One.

[250] Jin L. (2008). Mechanism of ubiquitin-chain formation by the human anaphase-promoting complex. Cell.

[251] Matsumoto M.L. (2010). K11-linked polyubiquitination in cell cycle control revealed by a K11 linkage-specific antibody. Mol Cell.

[252] Komander D. (2009). Molecular discrimination of structurally equivalent Lys 63-linked and linear polyubiquitin chains. EMBO Rep.

[253] Reinstein E. (2006). Narrative review: protein degradation and human diseases: the ubiquitin connection. Ann Intern Med.

[254] Terrell J. (1998). A function for monoubiquitination in the internalization of a G protein–coupled receptor. Mol Cell.

[255] Rome S. (2004). The ubiquitin-proteasome pathway is a new partner for the control of insulin signaling. Curr Opin Clin Nutr Metab Care.

[256] Mani A. (2005). The ubiquitin-proteasome pathway and its role in cancer. J Clin Oncol.

[257] Huang L. (1999). Structure of an E6AP-UbcH7 complex: insights into ubiquitination by the E2–E3 enzyme cascade. Science.

[258] Zheng N. (2002). Structure of the Cul1–Rbx1–Skp1–F boxSkp2 SCF ubiquitin ligase complex. Nature.

[259] Wu G. (2003). Structure of a β-TrCP1-Skp1-β-catenin complex: destruction motif binding and lysine specificity of the SCFβ-TrCP1 ubiquitin ligase. Mol Cell.

[260] Orlicky S. (2003). Structural basis for phosphodependent substrate selection and orientation by the SCF Cdc4 ubiquitin ligase. Cell.

[261] Hao B. (2005). Structural basis of the Cks1-dependent recognition of p27 Kip1 by the SCF Skp2 ubiquitin ligase. Mol Cell.

[262] Radivojac P. (2010). Identification, analysis, and prediction of protein ubiquitination sites. Proteins Struct Funct Bioinf.

[263] Chen X. (2013). Incorporating key position and amino acid residue features to identify general and species-specific ubiquitin conjugation sites. Bioinformatics.

[264] Qiu W.-R. (2015). iUbiq-Lys: prediction of lysine ubiquitination sites in proteins by extracting sequence evolution information via a gray system model. J Biomol Struct Dyn.

[265] Nguyen V.-N. (2016). UbiNet: an online resource for exploring the functional associations and regulatory networks of protein ubiquitylation. Database.

[266] Rodriguez M.S. (2001). SUMO-1 conjugation in vivo requires both a consensus modification motif and nuclear targeting. J Biol Chem.

[267] Sampson D.A. (2001). The small ubiquitin-like modifier-1 (SUMO-1) consensus sequence mediates Ubc9 binding and is essential for SUMO-1 modification. J Biol Chem.

[268] Kerscher O. (2007). SUMO junction—what's your function?. EMBO Rep.

[269] Geiss-Friedlander R. (2007). Concepts in sumoylation: a decade on. Nat Rev Mol Cell Biol.

[270] Hay R.T. (2005). SUMO: a history of modification. Mol Cell.

[271] Müller S. (2001). SUMO, ubiquitin's mysterious cousin. Nat Rev Mol Cell Biol.

[272] Seeler J.-S. (2003). Nuclear and unclear functions of SUMO. Nat Rev Mol Cell Biol.

[273] Lee L. (2013). SUMO and Alzheimer's disease. Neuromolecular Med.

[274] Zhao J. (2007). Sumoylation regulates diverse biological processes. Cell Mol Life Sci.

[275] Li M. (2005). SUMO wrestling with type 1 diabetes. J Mol Med.

[276] Blomster H.A. (2009). Novel proteomics strategy brings insight into the prevalence of SUMO-2 target sites. Mol Cell Proteomics.

[277] Veerbeek J.M. (2014). What is the evidence for physical therapy poststroke? A systematic review and meta-analysis. PLoS One.

[278] Impens F. (2014). Mapping of SUMO sites and analysis of SUMOylation changes induced by external stimuli. Proc Natl Acad Sci.

[279] Matic I. (2010). Site-specific identification of SUMO-2 targets in cells reveals an inverted SUMOylation motif and a hydrophobic cluster SUMOylation motif. Mol Cell.

[280] Zhao Q. (2014). GPS-SUMO: a tool for the prediction of sumoylation sites and SUMO-interaction motifs. Nucleic Acids Res.

[281] Beauclair G. (2015). JASSA: a comprehensive tool for prediction of SUMOylation sites and SIMs. Bioinformatics.

[282] Chen Y.-Z. (2012). SUMOhydro: a novel method for the prediction of sumoylation sites based on hydrophobic properties. PLoS One.

[283] Xu H.-D. (2015). Systematic analysis of the genetic variability that impacts SUMO conjugation and their involvement in human diseases. Sci Rep.

[284] Jia J. (2016). pSumo-CD: predicting sumoylation sites in proteins with covariance discriminant algorithm by incorporating sequence-coupled effects into general PseAAC. Bioinformatics.

[285] Beltrao P. (2013). Evolution and functional cross-talk of protein post-translational modifications. Mol Syst Biol.

[286] Strahl B.D. (2000). The language of covalent histone modifications. Nature.

[287] Meek D.W. (2009). Posttranslational modification of p53: cooperative integrators of function. Cold Spring Harb Perspect Biol.

[288] Xu Y.-X. (2003). Pin1 modulates the structure and function of human RNA polymerase II. Genes Dev.

[289] Westermann S. (2003). Post-translational modifications regulate microtubule function. Nat Rev Mol Cell Biol.

[290] Ivanov G.S. (2007). Methylation-acetylation interplay activates p53 in response to DNA damage. Mol Cell Biol.

[291] Estève P.-O. (2011). A methylation and phosphorylation switch between an adjacent lysine and serine determines human DNMT1 stability. Nat Struct Mol Biol.

[292] Ruan H.-B. (2013). Regulation of protein degradation by O-GlcNAcylation: crosstalk with ubiquitination. Mol Cell Proteomics.

[293] Bengoechea-Alonso M.T. (2009). A phosphorylation cascade controls the degradation of active SREBP1. J Biol Chem.

[294] Beltrao P. (2012). Systematic functional prioritization of protein posttranslational modifications. Cell.

[295] Minguez P. (2012). Deciphering a global network of functionally associated post-translational modifications. Mol Syst Biol.

[296] Pejaver V. (2014). The structural and functional signatures of proteins that undergo multiple events of post-translational modification. Protein Sci.

[297] Huang Y. (2015). Systematic characterization and prediction of post-translational modification cross-talk. Mol Cell Proteomics.

[298] Cornell W.D. (1995). A second generation force field for the simulation of proteins, nucleic acids, and organic molecules. J Am Chem Soc.

[299] Wang J. (2004). Development and testing of a general amber force field. J Comput Chem.

[300] Oostenbrink C. (2004). A biomolecular force field based on the free enthalpy of hydration and solvation: the GROMOS force-field parameter sets 53A5 and 53A6. J Comput Chem.

[301] Kaminski G.A. (2001). Evaluation and reparametrization of the OPLS-AA force field for proteins via comparison with accurate quantum chemical calculations on peptides. J Phys Chem B.

[302] Homeyer N. (2006). AMBER force-field parameters for phosphorylated amino acids in different protonation states: phosphoserine, phosphothreonine, phosphotyrosine, and phosphohistidine. J Mol Model.

[303] Han S. (2008). Force field parameters for S-nitrosocysteine and molecular dynamics simulations of S-nitrosated thioredoxin. Biochem Biophys Res Commun.

[304] Lu Z. (2009). Importance of charge independent effects in readout of the trimethyllysine mark by HP1 chromodomain. J Am Chem Soc.

[305] Machado M.R. (2010). Isoform-specific determinants in the HP1 binding to histone 3: insights from molecular simulations. Amino Acids.

[306] Grauffel C. (2010). Force field parameters for the simulation of modified histone tails. J Comput Chem.

[307] Woods R.J. (1995). Molecular mechanical and molecular dynamic simulations of glycoproteins and oligosaccharides. 1. GLYCAM_93 parameter development. J Phys Chem.

[308] Jo S. (2008). CHARMM-GUI: a web-based graphical user interface for CHARMM. J Comput Chem.

[309] Schmid N. (2011). Definition and testing of the GROMOS force-field versions 54A7 and 54B7. Eur Biophys J.

[310] Soares T. (2004). Validation of the GROMOS force-field parameter set 45A3 against nuclear magnetic resonance data of hen egg lysozyme. J Biomol NMR.

[311] Marrink S.J. (2007). The MARTINI force field: coarse grained model for biomolecular simulations. J Phys Chem B.

[312] de Jong D.H. (2013). Molecular view on protein sorting into liquid-ordered membrane domains mediated by gangliosides and lipid anchors. Faraday Discuss.

[313] Stewart-Jones G.B. (2016). Trimeric HIV-1-Env structures define glycan shields from clades A, B, and G. Cell.

[314] Olausson B.E. (2012). Molecular dynamics simulations reveal specific interactions of post-translational palmitoyl modifications with rhodopsin in membranes. J Am Chem Soc.

[315] Lakkaraju A.K. (2012). Palmitoylated calnexin is a key component of the ribosome–translocon complex. EMBO J.

[316] Zhou Q. (2014). Molecular insights into the membrane-associated phosphatidylinositol 4-kinase IIα. Nat Commun.

[317] Zoete V. (2011). SwissParam: a fast force field generation tool for small organic molecules. J Comput Chem.

[318] Vanquelef E. (2011). RED Server: a web service for deriving RESP and ESP charges and building force field libraries for new molecules and molecular fragments. Nucleic Acids Res.

[319] Malde A.K. (2011). An automated force field topology builder (ATB) and repository: version 1.0. J Chem Theory Comput.

[320] van Eerden F.J. (2015). Characterization of thylakoid lipid membranes from cyanobacteria and higher plants by molecular dynamics simulations. Biochim Biophys Acta Biomembr.

[321] Ingólfsson H.I. (2014). Lipid organization of the plasma membrane. J Am Chem Soc.

[322] Audagnotto M. (2016). Effect of the synaptic plasma membrane on the stability of the amyloid precursor protein homodimer. J Phys Chem Lett.

[323] van Eerden F.J. (2016). Molecular dynamics of Photosystem II embedded in the thylakoid membrane. J Phys Chem B.

[324] Bovigny C. (2015). LipidBuilder: a framework to build realistic models for biological membranes. J Chem Inf Model.

